# Lenvatinib in multimodal therapy for unresectable radioactive iodine‐naïve differentiated thyroid cancer: A case report with literature review

**DOI:** 10.1002/cnr2.1654

**Published:** 2022-06-17

**Authors:** Hugh Andrew Jinwook Kim, Anthony Charles Nichols, Ramanamurthy Rachakonda, Richard Inculet, Jinka Sathya, Irina Rachinsky, Eric Winquist

**Affiliations:** ^1^ Schulich School of Medicine and Dentistry University of Western Ontario London Canada; ^2^ Department of Otolaryngology – Head and Neck Surgery University of Western Ontario London Canada; ^3^ Department of Radiation Oncology Grand River Regional Cancer Centre Kitchener Canada; ^4^ Division of Thoracic Surgery, Department of Surgery University of Western Ontario London Canada; ^5^ Division of Radiation Oncology, Department of Oncology University of Western Ontario London Canada; ^6^ Department of Medical Imaging University of Western Ontario London Canada; ^7^ Division of Medical Oncology, Department of Oncology University of Western Ontario London Canada

**Keywords:** cancer care, cancer management, cancer medicine, clinical observations, head and neck cancer, immunotherapy

## Abstract

**Background:**

Patients with unresectable or metastatic differentiated thyroid carcinoma (DTC) are rare and require individualized therapy. This may require approaches not typically used in resectable disease. We report a patient treated with lenvatinib and external beam radiation therapy.

**Case:**

An 87‐year‐old woman presented with cT4N1aM1 papillary thyroid carcinoma with tracheal invasion. She was not a candidate for surgery, radioactive‐iodine, or radiation, so a trial of lenvatinib was offered. Her tumor showed clinical, biochemical, and radiological response after 5 months of lenvatinib, and she subsequently received external beam radiation. She enjoys good quality of life without evidence of cancer progression off therapy 21 months post‐initiation of treatment.

**Conclusion:**

Lenvatinib may be effective in RAI‐naïve advanced DTC patients as a component of individualized multimodal therapy when conventional options are not feasible.

## 1 BACKGROUND

Differentiated thyroid cancer (DTC) accounts for 3% of all cancer diagnoses in the United States.^1^ Five‐year survival rates in DTC range from nearly 100% in early‐stage disease to 51% in stage IV disease.^2^ Approximately 15% demonstrate extracapsular spread, and this tendency is greater in older patients with the papillary subtype.^3^ Papillary thyroid carcinoma is usually cured with thyroidectomy, but postoperative radioactive iodine (RAI) may offer improved survival and recurrence rates in cases with pathological risk factors or iodine‐avid distant metastases.^4^ For unresectable or metastatic RAI‐refractory disease, options to provide disease control and palliation include external beam radiation therapy (EBRT), chemotherapy, or targeted therapy.^5^ Vascular endothelial growth factor receptor tyrosine kinase inhibitors (TKIs) are effective in RAI‐refractory DTC; and sorafenib, lenvatinib, and recently cabozantinib (in the second‐line setting) have been studied in randomized trials and approved for use in these patients.^6^, ^7^, ^8^ Lenvatinib appears to be the most potent drug with a response rate of 64.8% (compared to 1.5% with placebo).^7^ With their consent, we report the case of a RAI‐naïve patient treated initially with lenvatinib followed by EBRT for locally advanced unresectable and metastatic papillary thyroid cancer and review the literature in this domain.

## 2 OBJECTIVE

To describe a case of sustained response to lenvatinib therapy in a patient with RAI‐naïve DTC.

## 3 CASE REPORT

**FIGURE 1:**
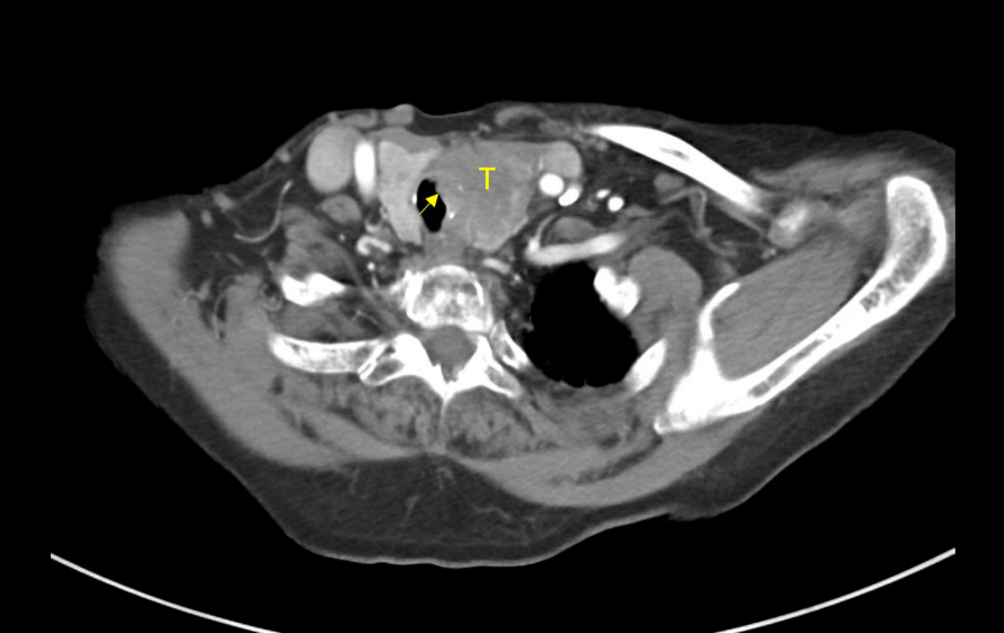
Locally advanced left thyroid tumor 2 months prior to initiation of lenvatinib therapy. T, tumor. Bulky tumor with invasion into the trachea is visualized (arrow).

An 87‐year‐old female was referred to our cancer center for management of locally advanced and metastatic papillary thyroid carcinoma. She reported a six‐month history of increasing stridor, shortness of breath, and hemoptysis, which prompted investigation with a biopsy of her left thyroid and computed tomography (CT) of the neck and chest. Imaging showed a 4.2 cm left thyroid mass indenting and displacing the cervical trachea, an enlarged left supraclavicular node, a 4.0 cm right adrenal mass, and bilateral pulmonary nodules suspicious for metastases (Figure 1). She was staged as cT4N1aM1, corresponding to stage IVB.

**FIGURE 2:**
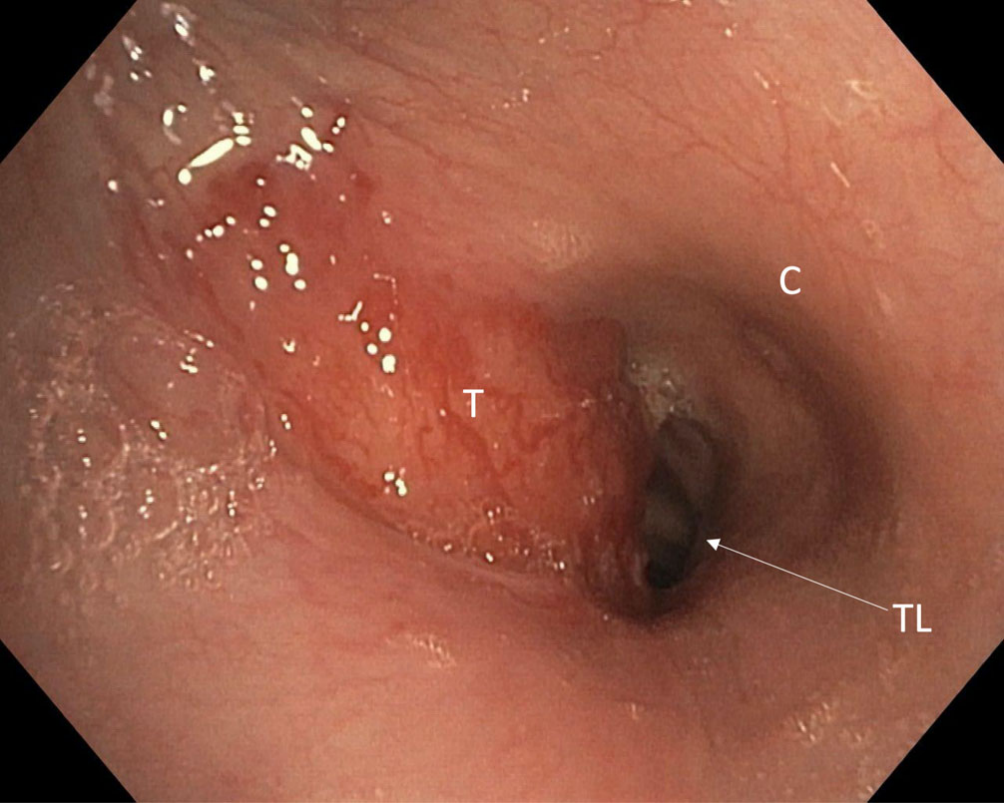
Endotracheal tumor extent visualized on flexible bronchoscopy. T, tumor; C, cricoid cartilage; Tr, tracheal lumen. Tumor is seen invading the left anterior tracheal wall and cricoid cartilage, decreasing the diameter by around 50%.

Flexible nasopharyngoscopy visualized a sluggish left vocal cord. Thoracic surgery assessment with flexible bronchoscopy visualized extensive tumor invasion of the left anterior tracheal wall involving the cricoid cartilage, decreasing the tracheal diameter by approximately 50% (Figure 2). It was presumed that without treatment, airway closure and death would follow within 2 months. Given the location of the tumor, surgical management would require total laryngectomy in addition to total thyroidectomy and central neck dissection.

The patient declined surgery due to her desire for voice preservation. She was not a candidate for RAI given her intact thyroid gland and nondebulked primary tumor. There was tepid enthusiasm for EBRT given the size of the treatment field. Lenvatinib was thus offered, with an understanding that it was being used under exceptional circumstances for a non‐approved indication, and the patient gave informed consent to proceed with therapy (provided by Eisai Limited, Mississauga, Ontario, Canada). In view of her age and health status, the starting dose was lenvatinib 14 mg p.o. daily with a plan to dose‐titrate based on tolerance.^9^

**FIGURE 3:**
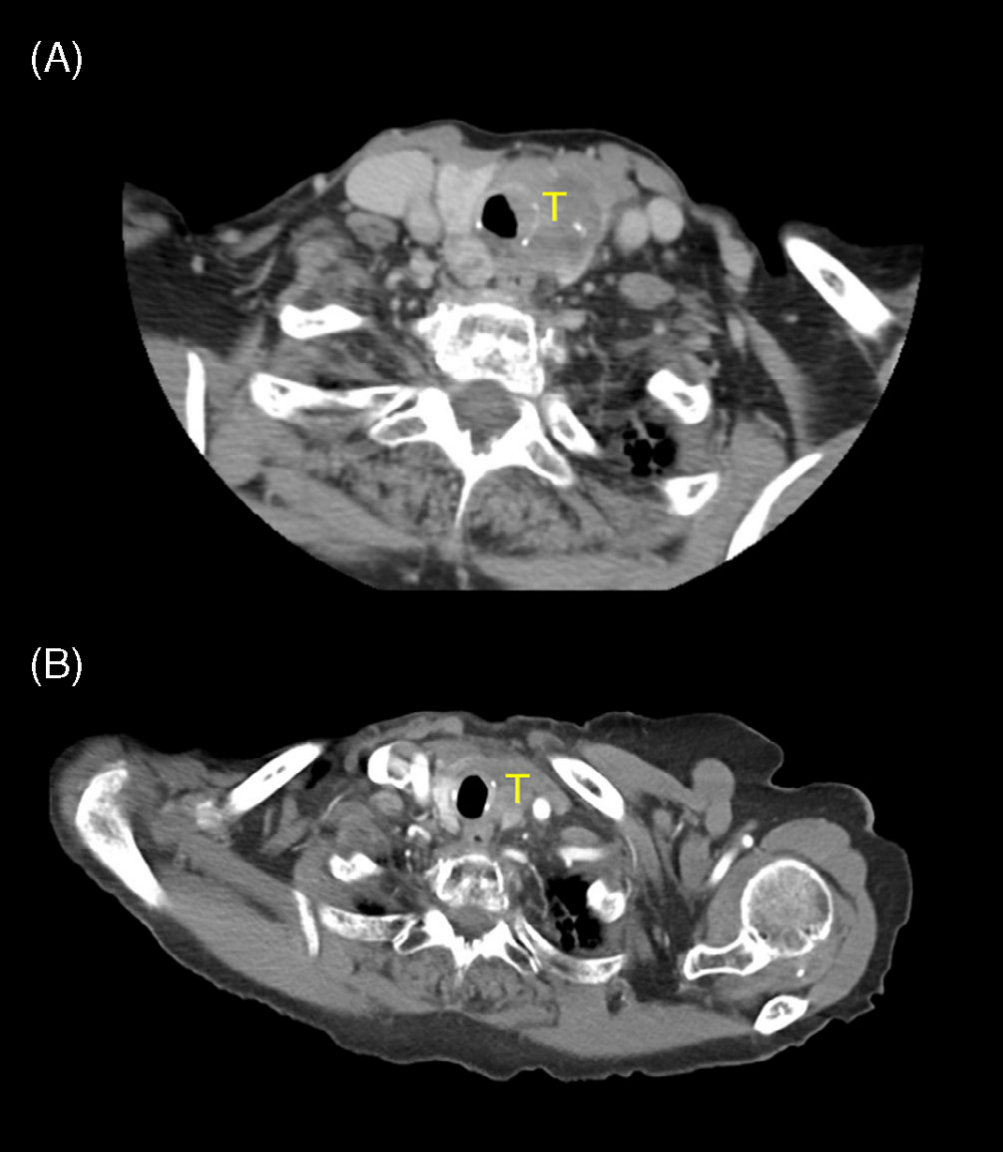
Locally advanced left thyroid tumor, (A) 3 months and (B) 16 months after initiation of lenvatinib therapy. T, tumor. The scan at 16 months includes effect from EBRT. Interval decrease in size is visualized at each scan.

Initially the patient was monitored weekly, and lenvatinib was well‐tolerated with gradual disappearance of stridor and dyspnea. After 1 month, treatment was interrupted for 2 weeks due to diverticulitis, then restarted at the same 14 mg dose. CT scan after 3 months of treatment demonstrated decrease in size of the left thyroid mass (2.9 cm), pulmonary nodules, and right adrenal mass (3.9 cm), compared to 2 months prior to treatment initiation (Figure 3A). Thyroglobulin rose from 54.6 μg/L to 408.9 ug/L and thyroid stimulating hormone (TSH) from 1.09 mIU/L to 6.72 mIU/L, and oral levothyroxine replacement 100 μg daily was initiated.

After 5 months of lenvatinib, local therapy was discussed in anticipation of eventual treatment resistance. The patient remained reluctant to pursue voice‐sacrificing surgery, and there had been no changes in recommendation regarding RAI, as the remnant normal thyroid remained large. Thus, 1 week after stopping lenvatinib, she received EBRT 50 Gy in 20 fractions to the thyroid, bilateral neck, and superior mediastinal nodes. She experienced moderately severe fatigue and radiation pharyngitis, which resolved over several months. Following EBRT, thyroglobulin was 25.7 μg/L and TSH 0.01 mIU/L, and CT showed stability of the thyroid mass (Figure 3B). In this context, lenvatinib was not restarted, and she has since been observed. At her last follow‐up 2 years after initiating lenvatinib, she was very satisfied with her quality of life and had resumed her usual activities with only mild residual dysphagia from EBRT.

## 4 DISCUSSION

The optimal therapeutic approach for the rare patient with unresectable and/or metastatic DTC is uncertain, may not be curative, and is usually individualized. Our patient was elderly, had metastatic and locally advanced disease threatening her airway, and refused surgery due to the need for laryngectomy. Given large unresectable tumor burden, RAI would have been ineffective and significantly delayed other potentially more effective therapies. This is because the expression of sodium iodide symporter necessary for RAI absorption is much more pronounced in normal thyroid tissue then in primary DTCs or metastases.^10^ For our patient, use of lenvatinib and subsequent EBRT resulted in rapid cancer control and resolution of symptoms with minimal side effects, with sustained response nearly 2 years from treatment initiation. In contrast, best supportive care alone would likely have resulted in airway compromise and death within 2 months.

A literature search for lenvatinib use in RAI‐naïve DTC identified six reports (Table 1). In all cases, patient or tumor factors initially made surgery and RAI too morbid or contraindicated, thereby prompting consideration of neoadjuvant lenvatinib to improve patient functional status or reduce tumor burden.^11^, ^12^, ^13^, ^14^, ^15^, ^16^ The patient who was managed most similarly to ours was treated nonsurgically with lenvatinib followed by EBRT and RAI, but unfortunately deteriorated secondary to malignant pleural effusion while receiving lenvatinib.^15^ In a retrospective study of TKI use in unresectable, RAI‐naïve DTC, the only patient who received first‐line lenvatinib had treatment withdrawn due to grade 3 asthenia, and another who received second‐line lenvatinib after pazopanib achieved stable disease.^17^ Our patient is unique in that lenvatinib, as a primary therapy rather than an adjunct, was well‐tolerated and demonstrated sustained radiological and clinical improvement in a frail non‐surgical patient. These results support consideration of lenvatinib as a component of individualized multimodality therapy for the uncommon patient with DTC in whom conventional surgery with adjuvant RAI is not feasible.

**TABLE 1:** Literature review of lenvatinib use in radioactive iodine‐naïve differentiated thyroid cancer

Study	Age/sex	Diagnosis	Non‐approved indication	Other treatments	Adverse events	Radiologic response (time interval)	Thyro‐globulin response (time interval)
Abhishek et al.^11^	77F	PTC vs FTC vs PDTC, M1	Palliative intent	Pre‐lenvatinib: docetaxel, cisplatin, 5‐fluorouracil Post‐lenvatinib: pazopanib	Atypical PRES, nephrotic syndrome	Tumor reduction in thyroid and liver (3 months)	NA
Garcia‐Rodriguez et al.^12^	41M	FTC, M1	ECOG score improvement	Pre‐lenvatinib: hemi‐thyroidectomy Post‐lenvatinib: completion thyroidectomy, RAI	Hypertension, diarrhea, stomatitis, neutropenia, palmar‐plantar erythro‐dysaesthesia	39.5% reduction (3 months)	49 105 to 2500 ng/ml (3 months)
Iwasaki et al.^13^	75F	PTC, M1	Urgent life‐saving treatment	Post‐lenvatinib: total thyroidectomy	Hypertension, PPE, anorexia	68 to 48 mm (4 months)	NA
Stewart et al.^14^	73F	PTC, pT4aN0M1	Declined surgery, RAI unindicated	Pre‐lenvatinib: sorafenib Post‐lenvatinib: hemi‐thyroidectomy, neck dissection, completion thyroidectomy	None	3.1 × 5.9 × 3.2 to 1.7 × 2.8 × 2.2 cm (14 months)	NA
Sukumar et al.^15^	69M	PTC, cT4aN0M0	Declined surgery, RAI unindicated	Pre‐lenvatinib: total thyroidectomy,paratracheal dissection, EBRT Post‐ lenvatinib: RAI	Myalgias, dysgeusia, weight loss	4.3 × 1.4 to 1.1 cm (14 months)	20 to 0.6 ng/mL (3 months)
	69F	PTC, cT4aN1bM1	Declined surgery, RAI unindicated	Post‐lenvatinib: EBRT, RAI	Hypertension, neuropathy, myalgias, weight loss	Tumor reduction in thyroid, lymph nodes, lungs; new malignant pleural effusion (15 months)	NA
Tsuboi et al.^16^	73M	PTC, cT4aN1bM0	Declined surgery, RAI unavailable	Post‐lenvatinib: total thyroidectomy, neck dissection, trachea‐esophageal resection	Proteinuria, hypertension	Lymph node reduction by 84.3% and 56.0%, primary reduction by 5.9% (6 months)	478 to 101 ng/mL (6 months)

*Notes*: Staging is based on AJCC 8th edition. Adverse events and response are reported as direct effects of lenvatinib.

Abbreviations: EBRT, external beam radiation therapy; ECOG, Eastern Cooperative Oncology Group; FTC, follicular thyroid carcinoma; PDTC, poorly differentiated thyroid carcinoma; PPE, palmar‐plantar erythro‐dysaesthesia; PRES, posterior reversible encephalopathy syndrome; PTC, papillary thyroid carcinoma; RAI, radioactive iodine.

## AUTHOR CONTRIBUTIONS

**Hugh Andrew Jinwook Kim:** Conceptualization (equal); visualization (equal); writing – original draft (equal); writing – review and editing (equal). **Anthony Charles Nichols:** Conceptualization (equal); writing – original draft (equal); writing – review and editing (equal). **Ramanamurthy Rachakonda:** Conceptualization (equal); writing – original draft (equal); writing – review and editing (equal). **Richard Inculet:** Conceptualization (equal); writing – original draft (equal); writing – review and editing (equal). **Jinka Sathya:** Conceptualization (equal); writing – original draft (equal); writing – review and editing (equal). **Irina Rachinsky:** Conceptualization (equal); writing – original draft (equal); writing – review and editing (equal). **Eric Winquist:** Conceptualization (equal); supervision (equal); visualization (equal); writing – original draft (equal); writing – review and editing (equal).

## CONFLICT OF INTEREST

Eric Winquist has served in a consulting/advisory role for Amgen, Bayer, Eisai, Ipsen, Merck, and Roche; and received research funding (institution) from Roche/Genenetech, Merck, Pfizer, Eisai and Ayala Pharmaceuticals. The other authors have no disclosures.

## ETHICS STATEMENT

Informed consent was obtained to use images and information from the patient whose case is published in this study.

## Data Availability

All data concerning this study are presented in this manuscript.
